# Effect of a CNS-Sensitive Anticholinesterase Methane Sulfonyl Fluoride on Hippocampal Acetylcholine Release in Freely Moving Rats

**DOI:** 10.1155/2012/708178

**Published:** 2012-01-29

**Authors:** Tamotsu Imanishi, Muhammad Mubarak Hossain, Tadahiko Suzuki, Ping Xu, Itaru Sato, Haruo Kobayashi

**Affiliations:** ^1^Department of Veterinary Medicine, Faculty of Agriculture, Iwate University, Ueda, Morioka 020-8550, Japan; ^2^Department of Food Safety, Pharmaceutical and Medical Safety Bureau, Ministry of Health, Labour and Welfare, Kasumigaseki, Chiyoda, Tokyo 100-8916, Japan; ^3^Department of Environmental and Occupational Medicine, Robert Wood Johnson Medical School, University of Medicine and Dentistry of New Jersey, 170 Frelinghuysen Road, Piscataway, NJ 08854, USA; ^4^Sciences of Cryobiosystems, United Graduate School of Agricultural Sciences of Iwate University, Ueda, Morioka 020-8550, Japan; ^5^7-272 Aza-Mukaishinden, Ukai, Takizawa-mura, Iwate-gun, Iwate Prefecture 020-0172, Japan

## Abstract

Anticholinesterases (antiChEs) are used to treat Alzheimer's disease. The comparative effects of two antiChEs, methanesulfonyl fluoride (MSF) and donepezil, on the extracellular levels of ACh in the hippocampus were investigated by *in vivo* microdialysis in freely moving rats. MSF at 1 and 2 mg/kg produced a dose-dependent increase in ACh efflux from 10 min to at least 3 hrs after injection. At 2 mg/kg, the increase was still present at 24 hr. Donepezil at 1 mg/kg showed a similar but smaller effect, and, paradoxically, 2 mg/kg showed no consistent effect. MSF at 1 and 2 mg/kg decreased acetylcholinesterase activity in the hippocampus to 54.8 and 20.1% of control, respectively. These results suggest that MSF is a suitable candidate for the treatment of Alzheimer's disease.

## 1. Introduction

Alzheimer's disease (AD) is a slowly progressive neurodegenerative illness characterized by the presence of senile plaques containing *β*-amyloid protein (A*β*) in brain tissue, tau-neurofibrillary tangles in neurons and, the loss of different transmitter-containing axons, especially cholinergic nerves [1, 2]. Unfortunately, therapeutic strategies targeting amyloid plaques with plaque-removing vaccines or gamma-secretase modulators have been disappointing [3, 4].

 It is generally accepted that progressive neurodegeneration of the cholinergic system underlies, at least in part, the cognitive deterioration of Alzheimer's disease (AD) [5–7]. This hypothesis is supported by findings of encouraging symptomatic improvements in clinical trials by the use of AChE inhibitors [8, 9], theoretically by enhancing central cholinergic function by permitting acetylcholine (ACh) to remain in the synaptic cleft longer. Interestingly, some AChE inhibitors have also been reported to be effective also in diminishing amyloid plaques [10, 11].

 Methanesulfonyl fluoride (MSF), a long-acting and highly specific inhibitor of brain AChE [12, 13], has been proposed as a safe and effective palliative treatment for senile dementia of the Alzheimer type [14] as well as a method to attenuate stroke-induced deficits in a simple learning and memory task [13]. Therefore, the main aim of this study was to compare MSF-induced increases in extracellular ACh in the hippocampus, one of the target regions for the treatment of AD, with the effects of donepezil, a reference drug widely used for symptomatic treatment of AD. For this purpose, the present study was carried out by measuring extracellular ACh in the hippocampus by *in vivo* microdialysis in freely moving rats following administration of MSF and donepezil.

## 2. Materials and Methods

Male Sprague-Dawley rats (Japan SLC, Hamamatsu, Japan) weighting 200–250 g were housed one per cage under the standard laboratory conditions (23 ± 1°C, 55 ± 5% humidity) with free access to standard pellet diet (MEQ, Oriental Yeast Co., Tokyo) and drinking water ad libitum with lights on at 08:00 and off at 20:00. Animal handling and procedures were conducted in accordance with the Animal Welfare Act and with the Guide for the Care and Use of Laboratory Animals approved by the Animal Experiment Committee in Iwate University, Japan. Five rats were used in each group.

 All reagents used were analytical grade. MSF was purchased from Sigma-Aldrich (Milwaukee, USA) and (*R,S*-1-benzyl-4-[(5,6-dimethoxy-1-indanon-2-yl)] methylpiperidine hydrochloride (donepezil) was a gift from Eisai Co., Ltd., (Tokyo, Japan).

 Microdialysis experiments were conducted according to Hossain et al. [15]. Briefly, the rats were anesthetized with sodium pentobarbital (50 mg/kg, i.p.) and then placed in a stereotaxic apparatus (Kopf instrument). The microdialysis guide cannula (AG-8, Eicom, Kyoto, Japan) was implanted into the left hippocampus with the following coordination (from the bregma): A*‒*5.8 mm, L+4.8 mm and, V*‒*4.5 mm. Following surgery, the animals were returned to their home cage and allowed to recover for at least 3 days before the beginning microdialysis. 

 ACh and choline content in the dialysate from the different animals were quantified by high-performance liquid chromatograph (HPLC) with electrochemical detection (ECD). The day of the experiment, the microdialysis probe (A-1-8-02, Eicom, Kyoto) was carefully inserted into the hippocampus through the guide cannula. The inlet of the microdialysis probe was connected to a 2.5 mL gastight syringe and perfused with Ringer's solution (NaCl 147 mM, KCl 4.0 mM and, CaCl_2_ 2.3 mM) containing 1 *μ*M eserine salicylate at a constant flow 2 *μ*L/min using a microperfusion pump, allowing the rats to move freely in a cubic Plexiglas box (30 cm × 30 cm × 40 cm).

 The dialysate collected during the first 30 min was discarded to ensure a stable baseline of ACh release. Thereafter, 20 *μ*L samples of perfusate were collected at 10 min intervals. Upon collection, 20 *μ*L of 1 *μ*M ethylhomocholine containing 10 mM EDTA 2Na was added to each sample as an internal standard. Levels of ACh and choline in the dialysate (20 *μ*L/injection) were determined by electrochemical detection with HPLC (Eicom, Kyoto, Japan) equipped with an enzyme column (AC-ENZ, Eicom, Kyoto, Japan). A 20 *μ*L sample of the perfusate/ethylhomocholine solution was then injected into a HPLC equipped with ECD (HPLC-ECD, Eicom, Kyoto) and enzyme column (AC-ENZ, Eicom, Kyoto).

 ACh and choline were separated on a cation exchange column (EICOMPAK AC-GEL, Eicom, Kyoto) with sodium lauryl sulfate (0.5 mg/mL). The mobile phase consisted of 0.05 M phosphate buffer (Na_2_HPO_4_ 12 H_2_O) pH 8.2 containing 0.13 mM EDTA 2Na, 0.6 mM tetramethylammonium chloride and, 1.2 mM SDS pumped at 1 mL/min. The retention times for choline and ACh were 7.3 and 13.2 min, respectively.

 The basal efflux was defined as the average output of three samples prior to drug administration, and the results were calculated as the percentage of the baseline choline and ACh.

 After establishing the basal efflux, the animals received one of the following IP injections: MSF (1 or 2 mg/kg) or donepezil (1 or 2 mg/kg) dissolved in vehicle (80 *μ*L ethanol + 88 *μ*L Tween 20) and prepared to a 1 mL total volume with isotonic sodium chloride. All control animals were injected with the same isotonic sodium chloride/vehicle solution by the same route and volume (1 mL/kg) as the drug.

 At the end of the microdialysis experiment, the rats were euthanized with chloroform, the brains were removed, and the position of the probe in the hippocampus was verified by visual examination of 20 *μ*m frozen sections.

 For acetylcholinesterase assays, rats in a parallel group received the same injections of MSF, donepezil, or vehicle on the same schedule as the animals used in microdialysis experiment. Three brain regions, hippocampus, striatum, and cerebral cortex (cortex) were quickly dissected on ice at 180 min and 24 hr after injection of MSF or donepezil and then homogenized in 0.1 M phosphate buffer solution (0.1 M Na_2_PO_4_+0.1 M KH_2_PO_4_, pH 8.0), followed by dilution with the same buffer to 200, 400, and 200 times of tissue weight, respectively, for the analysis of AChE activity by the method of Ellman et al. [16] with 0.48 mM acetylthiocholine iodide as substrate for 2 min at 25°C using UV-240 spectrophotometer (Shimadzu Corporation, Kyoto, Japan) at 412 nm.

 The extracellular levels of ACh and choline from individual rats were calculated relative to the mean basal release (the average of three 10 min sequential samples before drug administration was taken as 100% basal release). Analysis of variance, followed by Dunnett's post hoc test for repeated measurements (treatment versus time), was used to analyze changes from ACh and choline baselines as well as for tests of significant differences over time. A level of *P* < 0.05 was taken to indicate a statistically significant effect.

## 3. Results

The injections of MSF and donepezil (1 and 2 mg/kg i.p.) did not produce any observable clinical signs or symptoms in the rats. The basal rates of efflux from the hippocampus of vehicle-only injected control rats were 5.2 ± 0.2 pmol ACh/10 *μ*L/10 min and 180.8 ± 2.6 pmol choline/10 *μ*L/10 min (*n* = 15). The response of ACh in the hippocampus to vehicle treatment was not significantly different throughout the experiment.

**Figure 1:**
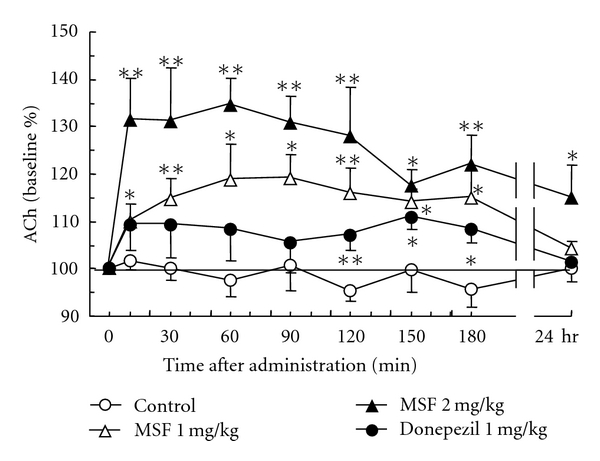
Effect of MSF and donepezil on the level of extracellular ACh in the freely moving rats. Data are expressed as percentage changes from baseline. Each value represents the mean ± S.E.M. of five experiments. Asterisks indicate effects significantly different from time course vehicle control (*P < 0.05, **P < 0.01).

As shown in Figure 1, MSF increased the release of ACh in a dose-dependent manner. MSF, at 1 mg/kg, caused a significant (*P* < 0.05, *P* < 0.01) and prolonged increase of ACh efflux in the hippocampus from 10 to 180 min which returned to control levels at 24 hr after the administration of MSF. At the higher dose of 2 mg/kg, MSF produced a consistent and proportionately larger increase in ACh release between 10 to 90 min (*P* < 0.01), decreasing progressively from 120 to 180 min (*P* < 0.05, *P* < 0.01) after the treatment. At 2 mg/kg MSF, the elevation of ACh efflux remained elevated at 24 hr after injection (*P* < 0.05).

 The effects of donepezil on ACh efflux are also shown in Figure 1. A dose of 1 mg/kg donepezil produced a small but consistent increase in ACh efflux over the first 180 minutes. However, the dose of 2 mg/kg did not produce a dose-dependent effect, and ACh efflux over the 180 min experiment was not different from animals received the dose of 1 mg/kg (data not shown).

**Figure 2:**
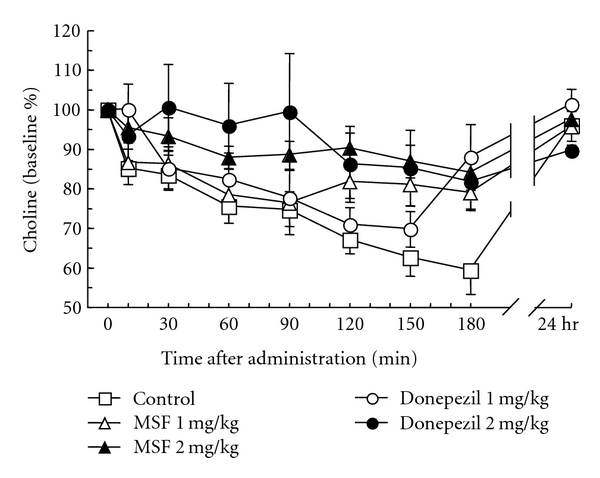
Effect of MSF and donepezil on the level of extracellular choline in the freely moving rats. Data are expressed as percentage changes from baseline. Each value represents the mean ± S.E.M. of five experiments.

 As shown in Figure 2, the choline efflux decreased progressively from basal levels throughout the course of the first 180 min after injection for every group, MSF, donepezil, and controls but returned to basal levels by 24 hr. The decreases were less significant in the groups that received MSF or donepezil than in the control group.

**Figure 3:**
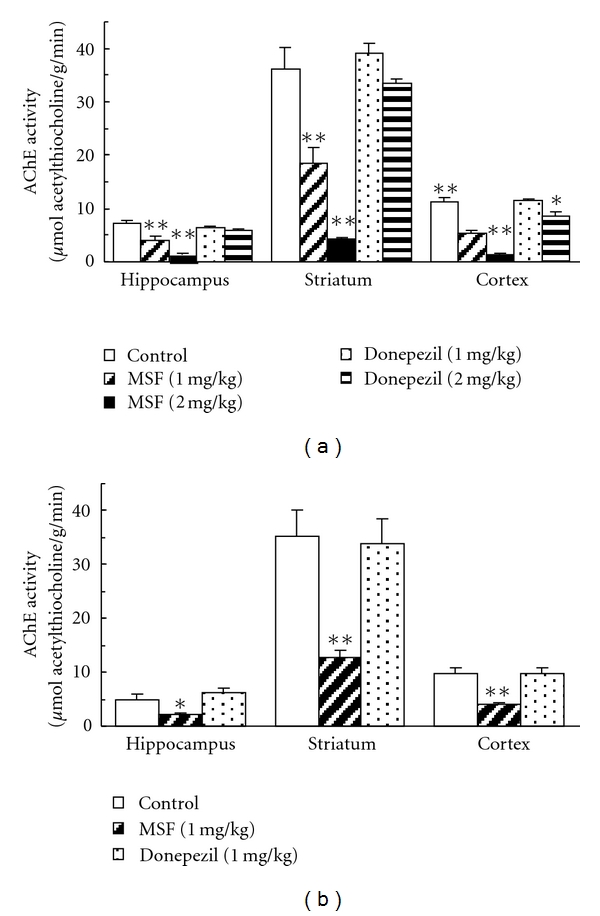
Effect of MSF and donepezil on the activity of AChE in brain regions 180 min (a) and 24 hr (b) after administration. Each value represents the mean ± S.E.M. of five experiments. Asterisks indicate effects significantly different from time course vehicle control (*P < 0.05, **P < 0.01).

 The effect of MSF and donepezil on AChE activity in the hippocampus, striatum, and cortex at 180 min (Figure 3(a)) and 24 hr (Figure 3(b)) after drug administration is shown in Figure 3. MSF at 1 mg/kg decreased AChE activity in hippocampus, striatum, and cortex by about 50%, to 55, 51, and 49% of the respective control activities. At 2 mg/kg, MSF produced 80–90% inhibition, bringing AChE activity to about 20, 12, and 11% of the respective control activities. Donepezil at 1 mg/kg did not produce any significant effect on AChE activity in the three brain regions at 180 min after injection. The dose of 2 mg/kg, however, produced a significant decrease in AChE activity in the cortex but not in the hippocampus or striatum 180 min after administration (Figure 3(a)). Figure 3(b) also shows the brain regional AChE activity 24 hr after 1 mg/kg of MSF or donepezil. The activity of AChE in the hippocampus, striatum, and cortex of rats administered MSF was about 44, 36 and 41% (*P* < 0.05 to 0.001) of control values, respectively. At 24 hr, no other significant differences were not observed in the activity of AChE in any of the three brain regions between the rats treated with donepezil or vehicle.

## 4. Discussion

We have previously reported that a single dose of MSF at 1.5 mg/kg s.c. significantly increased the concentrations of extraterminal ACh and cytoplasmic ACh in the cortex of mice [17]. In these experiments, an increase in the fractional ACh content of brain tissues taken *ex vivo* and homogenized for analysis was found to be elevated at 180 min, and the increase persisted to 24 hr. The extraterminal ACh determined in that earlier *ex vivo* experiment may approximately correspond to extracellular ACh in the current *in vivo* experiment at those same time points. The present experiment confirmed the earlier results and showed that doses of either 1 mg/kg or 2 mg/kg MSF strongly increase extracellular ACh during the first 180 min after administration and the effect persists for 24 hr after the higher dose of 2 mg/kg.

 Corresponding to the increases in ACh efflux found after MSF, the present study also found that the same doses reduced AChE activity in the hippocampus to about 55% and 20% of control 180 min after 1 mg/kg and 2 mg/kg of MSF, respectively, and to about 44% of control 24 hr after 1 mg/kg of MSF (the only dose studied at 24 hr). These results support several studies on the effects of drugs for Alzheimer's disease that show that increasing levels of extracellular ACh in various brain regions are found with decreasing AChE activity [8, 9, 18–22].

 Donepezil (1.66 mg/kg), rivastigmine (0.4 mg/kg), and huperzine A (more than 0.6 and 3 mg/kg) have been found to increase extracellular ACh in the hippocampus, and the results are generally not so different between the oral or intraperitoneal administration [8, 18]. All three of these drugs produced the maximal increase in extracellular ACh within 3 hrs, mostly around 30 to 60 min, after administration. On the other hand, the present study shows that MSF increased the level of extracellular ACh for at least 3 hrs without showing significant peaks. After 2 mg/kg MSF, a significant increase was still present at 24 hr.

 Although donepezil is generally accepted to be effective in increasing the release of ACh from hippocampus in freely moving animals [8, 9], the present study, however, found that donepezil succeeded in increasing extracellular ACh only at a dose of 1 mg/kg. Surprisingly, it failed to demonstrate a dose-dependent effect at a dose of 2 mg/kg. Although the doses of donepezil, 1 mg/kg and 2 mg/kg, were selected for comparison to the MSF results, a possible explanation for our failure to find a dose-dependent effect of donepezil on extracellular ACh may be that the dose of 2 mg/kg was not sufficiently greater than the 1 mg/kg dose to produce a clear difference. The dose-dependent effects of donepezil on increasing the level of extracellular ACh in the striatum achieved by previous reports [18] were obtained by using doses at 1, 3, and 5 mg/kg.

 In the present study, donepezil at doses of 1 and 2 mg/kg did not show any significant inhibitory effect on the activity of AChE in the hippocampus at 180 min after administration (Figure 3(a)). Donepezil is a reversible AChE inhibitor [9, 23] and the AChE inhibition it produces may disappear when the tissues are homogenized as the donepezil will be diluted.

## 5. Conclusions

The present study showed that MSF at doses 1 and 2 mg/kg produced a consistent increase in the efflux of ACh in freely moving rats as measured by microdialysis throughout the first 3 hrs at both doses and a persistent increase was still present at 24 hr at the higher dose. Since ChE inhibitors are the major therapeutic agents used in AD patients, the agent-like MSF, which increases extracellular ACh in the hippocampus with a long-lasting efficacy but without excess stimulation, may serve as an effective therapy to alleviate or prevent the central cholinergic deficits which are reported to cause cognitive impairments.
